# Editorial: COVID-19 pandemic, food behaviour and consumption patterns

**DOI:** 10.3389/fpubh.2022.1039419

**Published:** 2022-10-24

**Authors:** Tarek Ben Hassen, Hamid El Bilali, Mohammad S. Allahyari, Sinisa Berjan

**Affiliations:** ^1^Program of Policy, Planning, and Development, Department of International Affairs, College of Arts and Sciences, Qatar University, Doha, Qatar; ^2^International Centre for Advanced Mediterranean Agronomic Studies, Bari, Italy; ^3^Department of Agricultural Management, Islamic Azad University, Rasht, Iran; ^4^Faculty of Economic and Management Sciences, North-West University, Mmabatho, South Africa; ^5^Department of Agroeconomy and Rural Development, Faculty of Agriculture, University of East Sarajevo, Lukavica, Bosnia and Herzegovina

**Keywords:** COVID-19 pandemic, food behavior changes, food security, consumption patterns, food safety

With already 600 million confirmed cases of COVID-19 and over 6 million recorded deaths, the Coronavirus Disease 2019 (COVID-19), detected in Wuhan (China) in late 2019, is nowadays one of the most pressing global challenges facing humanity. In addition to significantly impacting health systems, the COVID-19 pandemic disrupted food systems from farm to fork, with consequences for food and nutrition security at all levels (global, national, local, and individual). While a growing corpus of research examines the pandemic's disruption of food supply networks, the implications regarding food environments and consumer behavior are still widely overlooked, particularly in developing countries. Accordingly, this Research Topic intends to offer insight into the pandemic's influence on food buying behavior, nutrition, and eating habits and the consequences of these changes. It includes 10 papers on various issues (diet, food security, food affordability, food safety, shopping habits, food waste, etc.) and geographical areas (Oman, Jordan, Saudi Arabia, Italy, Canada, and India).

Regarding the influence of the pandemic on diet and food choices, in their study in Oman, Ben Hassen et al. highlighted a significant change in the attitude and behavior of respondents regarding food and health, such as a shift to healthier diets, an increase in the consumption of local products, buying more groceries online; and a reduction of food waste. Further, Vetrani et al. examined the impact of the lockdown on eating habits in individuals in Italy with type 1 diabetes (T1D) on a hybrid artificial pancreas (HAP). They reported that Italian patients with T1D on HAP altered their eating choices during the lockdown by consuming less animal protein and more carbohydrates. This increase in whole grain and low-glycemic index items did not affect blood glucose control.

Additionally, in their longitudinal study, Alshahrani et al. assessed the impact of the COVID-19 pandemic on body weight and body mass index (BMI) in Saudi Arabia. They pointed out that about one-quarter (23%) of their sample had gained 5% or more of their pre-2020 weight. Females saw more weight increases from pre-2020 to post-2020. These data highlight COVID-19's negative externalities in terms of its influence on infections and other health disorders that affect population health.

Furthermore, Nielsen et al. showed that COVID-19 affected food purchases in Quebec, Canada. In-store food purchases were lowest during the lockdown (once a week or less), then rose to pre-pandemic levels. During the lockdown, concerns regarding grocery store virus exposure and disinfection/disposal of food packaging peaked. Frequent usage of no-contact grocery methods was linked to public transit, walking or cycling.

In developing countries, the impact of the pandemic on food security was significant.  Padmaja et al. studied the pandemic's effects on food security and coping methods in Hyderabad, India. The findings showed over 40% of families interviewed observed food security decline during the pandemic. It also showed that food security was strongly linked to a family's main income earner's sector of activity. The surveyed households adopted different consumption-smoothing strategies, including borrowing from official and informal sources and liquidating savings. Along the same line of inquiry, Jeyakumar et al. studied food availability, accessibility, and affordability during the first wave of the pandemic in selected districts of Chhattisgarh in India. Of the 63% non-tribal population, a more significant percentage experienced income loss (13.4%) and worried about not having sufficient food (40%). Non-tribal areas reported more food shortages (34%) and hunger (15%) than tribal areas. To overcome the pandemic's consequences, immediate and vulnerable-focused interventions must be addressed.

Regarding food safety, Osaili et al. evaluated food safety knowledge, attitudes, and practices (KAP) amongst university students in Jordan and changes in food-related behaviors during the COVID-19 pandemic. They concluded that university students in Jordan have insufficient food safety knowledge. They recommended that the basics of food safety be taught through short courses/lectures. Further, Luo et al. analyzed the relevant literature on food safety in the food supply chain and assessed its present state, hot spots, and development patterns. They concluded that future food supply chain management might become an important topic, particularly when traceability management and the blockchain are linked.

Regarding virus transmission, Rafieepoor et al. investigated the presence of SARS-CoV-2 virus RNA along the food production and retail chain in Tehran (Iran), from wastewater to water used for irrigation and harvested and marketed vegetables. The results revealed that SARS-CoV-2 was prevalent in retail food and could potentially contaminate agricultural water and products. This research showed that although SARS-CoV-2 RNA was identified on minimally washed and raw foods, there was no indication of a public health risk.

Finally, according to Maffetone and Laursen, the COVID-19 pandemic was a predictable and avoidable disaster. It should serve as a wake-up call to public health and healthcare professionals, politicians, and citizens. While many reactive measures to the pandemic were implemented, preventative efforts were overlooked in the years before COVID-19. They recommended implementing proactive lifestyle changes with basic, safe, and affordable dietary improvements that may lead to a healthier population.

The results of the studies presented in this Research Topic confirm that the consequences of COVID-19 vary by country, based not only on the epidemiological situation but also, among other factors, on the baseline socio-economic situation and level of resilience to shocks. Furthermore, since there is no widely available literature on modern pandemics other than SARS, these studies help to provide a better understanding of future shocks and crises and their potential impact. Current and future research will serve as a foundation for organizational and government readiness and preparedness for future public health shocks, including new pandemics.

## Author contributions

All authors listed have made a substantial, direct, and intellectual contribution to the work and approved it for publication.

## Conflict of interest

The authors declare that the research was conducted in the absence of any commercial or financial relationships that could be construed as a potential conflict of interest.

## Publisher's note

All claims expressed in this article are solely those of the authors and do not necessarily represent those of their affiliated organizations, or those of the publisher, the editors and the reviewers. Any product that may be evaluated in this article, or claim that may be made by its manufacturer, is not guaranteed or endorsed by the publisher.

